# Integrative analysis of bulk and single-cell transcriptomics identifies factors related to immunosuppressive microenvironment to predict unfavorable prognosis in inflammatory breast cancer

**DOI:** 10.3389/fimmu.2025.1727590

**Published:** 2026-01-06

**Authors:** Kexuan Feng, Xiaoduo Li, Xiaoqian Li, Junjie Liu, Rui Zhang, Heyan Chen, Yunpeng Zhang, Jianjun He, Huimin Zhang

**Affiliations:** 1 Department of Breast Surgery, The first Affiliated Hospital of Xi’an Jiaotong University, Xi’an, Shaanxi, China; 2 Department of Breast Surgery, Fujian Medical University Union Hospital, Fuzhou, Fujian, China

**Keywords:** immunosuppressive microenvironment, inflammatory breast cancer, plasmacytoid dendritic cells, prognostic biomarker, single-cell transcriptomics

## Abstract

**Introduction:**

Inflammatory breast cancer (IBC) is a rare invasive tumor and characterized by the formation of tumor emboli within dermal lymphatic vessels. The tumor microenvironment (TME) is a key factor in IBC aggressiveness, but its heterogeneity and intercellular role remain incompletely understood.

**Methods:**

Weighted gene coexpression network analysis (WGCNA) was performed to identify immune infiltration-associated genes. We also depicted cellular communication networks and specific ligand-receptor signaling pathways using the single-cell transcriptomics analysis of IBC and non-IBC samples. Finally, we verified the expression of key proteins by immunohistochemistry.

**Results:**

Through the intersection of WGCNA module genes and differentially expressed genes between pathological complete response and residual disease samples, *GZMB* was identified as hub gene which is related to immune infiltration and efficacy of neoadjuvant therapy in IBC. In IBC cohort, patients with high expression of *GZMB* harbored more immunosuppressive cells thus showed unfavorable prognosis compared with *GZMB*-low expression group (p<0.05). Subsequent to dimension reduction and clustering, 12 clusters were identified to construct the single-cell atlas between IBC and non-IBC samples. Cellular communication analysis unveiled the heterogeneity of cell communication in IBC. The proportion of immune cells was significantly lower than that of malignant epithelial cells in the cellular composition of IBC. Moreover, it indicated that *SPP1* and plasmacytoid dendritic cells were specific in IBC and associated with an immunosuppressive microenvironment in IBC. Immunohistochemical analysis suggested that protein levels of *GZMB* and *SPP1* tended to be higher in the samples from patients with residual disease compared to the patient achieving pathological complete response, though this observation is based on an extremely small sample size.

**Discussion:**

This study identifies GZMB and SPP1 as potential immunosuppressive-related prognostic biomarkers in IBC patients, reveals the key role of plasmacytoid dendritic cells in remodeling of immunosuppressive microenvironment in IBC.

## Introduction

IBC is a highly aggressive disease and characterized by the formation of tumor emboli within dermal lymphatic vessels (1). It accounts for 2-4% of all breast cancer cases in the U.S. and contributes to 7-10% of breast cancer-related deaths in western countries (2). The American Joint Committee on Cancer (AJCC) clinical staging criteria categorize IBC as T4d. Despite the incidence of IBC is low, the prognosis is very poor (3). The reported median overall survival (OS) of patients with IBC compared to those with non-IBC breast cancer is 4.75 years versus 13.40 years for stage III disease (4) and 2.27 years versus 3.40 years for stage IV disease (5). Current treatment for IBC remains similar to non-IBC which was guided by molecular subtype, including targeted therapy for HER2+ patients, chemotherapy and immunotherapy for triple negative breast cancer patients, and endocrine therapy for HR+ patients (6, 7). For IBC, neoadjuvant therapy (NAT) were recommended, mastectomy was standard of care. Pathological complete response (pCR) to NAT is associated with improved progression-free survival and OS (8), but IBC responds poorly to NAT compared to non-IBC, with a pCR rate of only 15.2% (9). Due to the lack of specific treatment, the 5-years survival rate for IBC remains below 50% (10) and the identification of new therapeutic targets is critical.

Previous studies have shown that the TME is a key factor in the aggressiveness of IBC and correlates with chemoresistance and unfavorable prognosis (11–13). For instance, compared with non-IBC, differences in the composition of resident cell types within the TME, including dendritic cells (DCs), tumor-associated macrophages (TAMs), mesenchymal stem cells and fat stem cells as well as associated inflammatory pathways have been reported in IBC (11, 14). Nevertheless, the study on TME-related biomarkers which can predict clinical outcomes of IBC is limited, and the combined impact of intercellular communication and signaling pathway activation in microenvironment remodeling and tumor development remains unclear.

The present study sought to investigate regulatory mechanisms within the TME that may underlie the aggressive behavior of IBC. By integrating bulk and single-cell transcriptomic analyses complemented by immunohistochemical validation, study demonstrated that *GZMB* and *SPP1* contribute to an immunosuppressive TME and are significantly associated with resistance to NAT and poor prognosis in IBC. Through single-cell analysis, study compared the differences in intercellular communication between IBC and non-IBC and further identified plasmacytoid dendritic cells (pDCs) as a IBC-specific cellular subset promoting tumor aggressiveness. Collectively, this study delineates key biomarkers and cellular mediators of immune dysregulation in IBC, elucidates TME remodeling processes, and establishes *GZMB* and *SPP1* as potential prognostic biomarkers for IBC patients.

## Materials and methods

### Patients and datasets

This study gathered 102 IBC samples and 2 non-IBC samples from GEO database (https://www.ncbi.nlm.nih.gov/geo/). For the discovery cohort, the RNA sequencing (RNA-seq) data and corresponding clinical information of 43 IBC samples were collected from GSE17907 and GSE207248 datasets. To assess the generalizability and specificity of the finding, we extracted TCGA-BRCA samples(n=1079) with complete clinical information from the UCSC Xena database (https://xena.ucsc.edu) as the model validation set. The RNA-seq data and clinical information of 79 samples were downloaded from GSE5847, GSE17907, GSE22597, and GSE45581 datasets for the validation cohort. For single-cell analysis, the single-cell RNA sequencing (scRNA-seq) data and related clinical information of 1 IBC sample and 2 non-IBC samples were obtained from GSE208532. All data generated or analyzed during this study are freely available in previous publications or the public database (**Supplementary Table 1**).

### Computation of immune infiltration

ESTIMATE was used to evaluate the immune cell and stromal content for each sample (15). Single-sample gene-set enrichment analysis (ssGSEA) was performed to quantify the enrichment levels of immune signatures with R package GSVA (16).

### WGCNA for module identification and differentially expressed gene analysis

**Figure 1:**
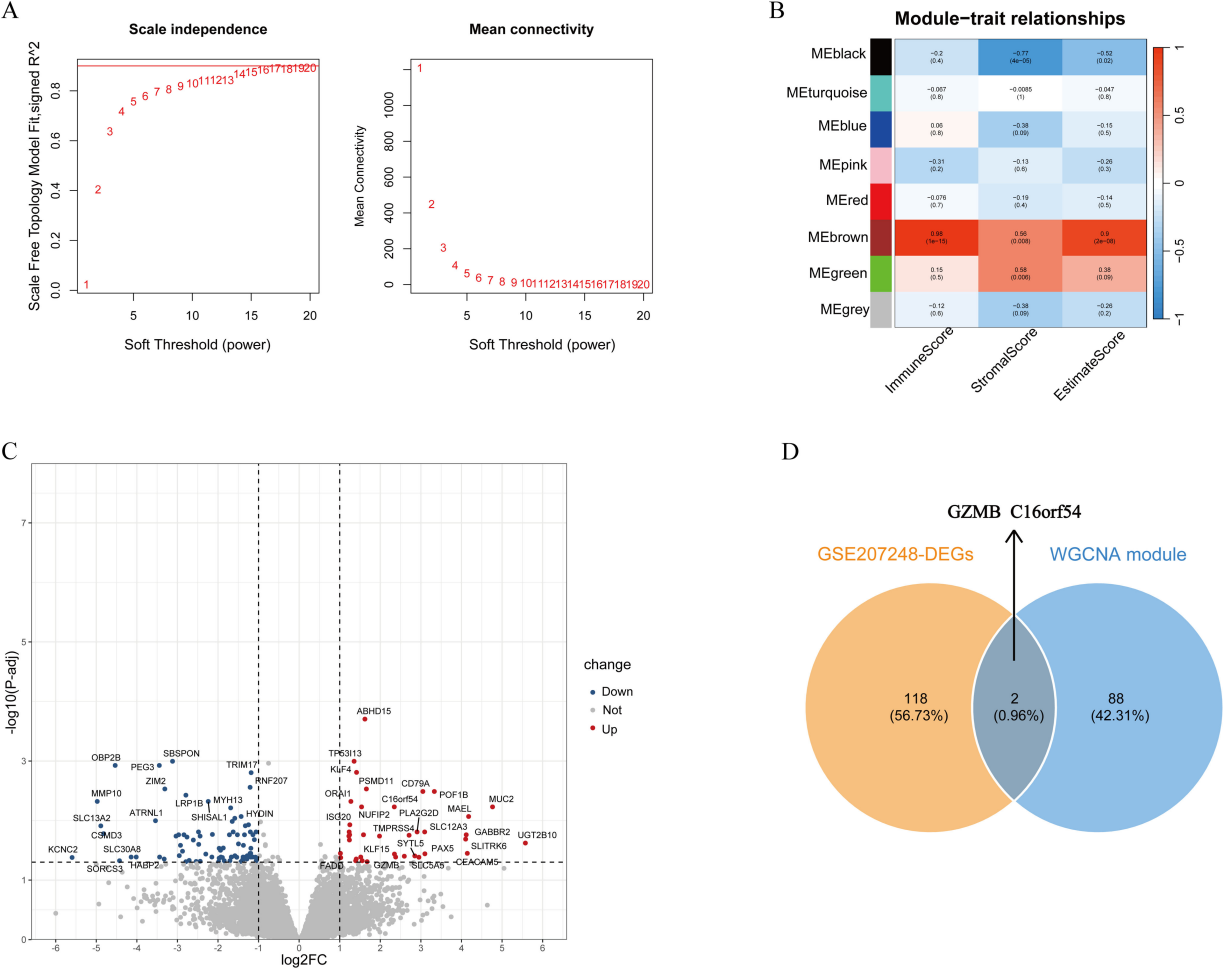
Screening for hub genes linking tumor immune infiltration to response to NAT. **(A)** Selection of the soft-thresholding power (β). Left panel: The scale-free topology fit index (R^2^) across different powers. The red dashed line indicates the threshold used for selection. Right panel: The corresponding mean connectivity (log10 scale). The chosen power β = 8. **(B)** Heatmap of correlation between different modules and immune scores. **(C)** Volcano plot of DEGs between pCR and RD samples. **(D)** Venn plot showing the hub genes intersected by WGCNA and DEGs.

Weighted gene co-expression networks were constructed using the WGCNA R package (v1.72-5). After filtering lowly expressed genes, the optimal soft-thresholding power (β) was determined using the pickSoftThreshold function. The lowest power that achieved a scale-free topology fit index (R^2^) > 0.9 was selected (β = 8, see **Figure 1A**). This adjacency matrix was transformed into a Topological Overlap Matrix (TOM), based which gene modules were identified via hierarchical clustering with dynamic tree cutting. Additionally, DEG analysis was performed using the DESeq2 package, where genes with a P-adjust<0.05 and |log2 (fold change (FC)) |>1 were designated as DEGs. The hub genes emerged through the intersection of immune infiltration-related gene sets and DEGs.

### Survival prediction and predictive performance of hub gene

IBC cohort was divided into hub gene high and low expression group based on the median cutoff value. Study performed K-M survival analysis and visualization using the survival and survminer packages to compare prognosis between hub gene high and low expression group. In addition we evaluated predictive performance of hub gene through time-dependent receiver operating characteristic(ROC) analysis. The analysis and visualization were conducted using R packages, including pROC, ggplot2, survival, survminer, and timeROC.

### Drug response prediction

We mainly used a ridge regression-based method of the R package oncoPredict (version 1.2) to predict the half maximal inhibitory concentration (IC50) values of potential drug responses. The transcriptomic data of cell lines from CCLE and drug response data from CTRP were utilized as training sets. The transcriptomic data of IBC samples were utilized as testing sets. We used the calcPhenotype function to calculate the IC50 values for each corresponding drug.

### Processing of scRNA-seq data

We mainly used Seurat (version 5.1.0) to perform the single-cell analysis. Samples with over 10% mitochondrial genes were excluded. We also filtered out the genes expressed in fewer than three cells, and cells with fewer than 200 or more than 5000 expressed genes. Following these stringent quality control criteria, a total of 18,834 high-quality cells were retained for all downstream analyses. The FindVariableFeatures function in Seurat identified the most variable genes. Principal Component Analysis was utilized for dimensionality reduction. The harmony R package was employed to remove the batch effects. A total of 12 clusters were identified by uniform manifold approximation and projection (UMAP) cluster analysis, and cell types were finally annotated by combining reported and most changed marker genes.

### DEG analysis and gene enrichment analysis

DEGs of different cell types were identified through the FindAllMarkers function of Seurat using the wilcox test and the fold change with the value 2. We used the R package clusterProfiler (version 4.10.0) to perform Kyoto Encyclopedia of Genes and Genomes (KEGG) enrichment analysis, therefore determining the biological and molecular functional processes of the prioritized gene list as well as their significantly enriched pathways. All hallmark gene sets were obtained from The Molecular Signatures Database.

### CellChat analysis

To discover potential intercellular communications based on ligand-receptor interactions, we used the R package CellChat (version 2.1.2), which provides ligands, receptors, and cofactors and computes their interactions to build cell-cell communication atlases. The normalized expression matrix, which was annotated by Seurat, was imported, and a CellChat object was created with the createCellChat function. After preprocessing data followed the standard workflow with the identifyOverExpressedGenes function, identifyOverExpressedInteraction function, projectData function, computeCommunProb function, computeCommunProbPathway function, filterCommunication function and aggregateNet function, the aggregated cell-cell communication networks of these cell types were calculated.

### Pseudotime analysis

To investigate potential differentiation trajectories of myeloid cells within the inflammatory breast cancer microenvironment, we extracted all cells annotated as “myeloid/monocytes” from the integrated and annotated single-cell dataset, forming a subset of 75 myeloid/monocytes for subsequent analysis. We performed single-cell pseudotemporal trajectory analysis using the R package Monocle2 (version 2.32.0) with the DDR-Tree method and default parameters (17). The log-normalized expression data from this myeloid cell subset was used as the input. Differentially expressed genes or marker gene sets from the cell clusters were applied as the ordering gene sets. Cell trajectories were inferred following dimensionality reduction and cell ordering using the default parameters.

### Immunohistochemistry

Paraffin-embedded breast tissues of 3 pre-treatment IBC patients from our institution were collected for immunohistochemical staining (**Supplementary Table 2**). In brief, the sections were incubated with antibodies overnight at 4 °C, and then with secondary antibodies at room temperature for 50 minutes. After staining with DAB (Servicebio, Wuhan, Hubei, PR China) for 1 minute, sections were counterstained with hematoxylin (Servicebio, Wuhan, Hubei, PR China). The used antibodies included anti-*GZMB* (Servicebio, GB15092, 1:1000) for *GZMB* and anti-*SPP1* (Servicebio, GB11500, 1:1000) for *SPP1*. All antibodies were validated by the commercial vendor. 10 fields of each section were selected and the histochemical scoring method (H-score) was employed for quantitative analysis.

### Statistical analysis

All statistical analyses were performed using R software (version 4.3.1) and GraphPad Prism (version 10.1.2). For bulk transcriptomic data from the GSE5847 cohort, the association of gene module eigengenes or individual gene expression (e.g., GZMB) with overall survival was evaluated using the Kaplan-Meier method and compared with the log-rank test. Hazard ratios (HRs) and 95% confidence intervals (CIs) were derived from univariate Cox proportional hazards regression. Differential gene expression between IBC and non-IBC groups was analyzed using the limma R package. Weighted gene co-expression network analysis (WGCNA) was performed to identify modules correlated with clinical traits. For the validation cohort (TCGA-BRCA), the prognostic value of *GZMB* was similarly assessed using Kaplan-Meier survival analysis and Cox regression. Single-cell RNA-seq data were processed and analyzed using the Seurat R package (version 4.3.0). Differential expression analysis between cell types or between IBC and non-IBC samples within a specific cell lineage was performed using the Wilcoxon rank-sum test. Cell-cell communication networks were inferred using the CellChat R package (version 1.6.0), which employs a probabilistic model to identify over-represented ligand-receptor interactions. For comparisons among more than two groups, one-way ANOVA (with Tukey’s post-hoc test) or the Kruskal–Wallis test (with Dunn’s post-hoc test) was applied as appropriate. Correlation analyses were conducted using Pearson’s correlation coefficient for linear relationships or Spearman’s rank correlation coefficient for monotonic relationships. A two-sided P-value < 0.05 was considered statistically significant unless otherwise specified.

## Results

### Screening for hub genes linking tumor immune infiltration to response to NAT

To investigate the role of the immune microenvironment in the tumorigenesis and progression of IBC, study applied the ESTIMATE algorithm to compute immune scores for a cohort of 21 pre-treatment IBC samples from GSE17907 dataset. Clinical characteristics of the 21 samples are summarized in **Supplementary Table 3**. Study subsequently employed WGCNA to identify gene modules associated with immune infiltration, which involved constructing co-expression networks and defining co-expression modules (**Figure 1A**). Correlations between immune scores and module eigengenes were assessed using Pearson’s correlation tests. The brown module, comprising 90 genes, was most significantly correlated with immune infiltration (**Figure 1B**). Additionally, study performed DEG analysis on 22 pre-treatment IBC samples who received NAT from GSE207248 dataset to identify genes differentially expressed between pCR and residual disease(RD) groups, and yielded 120 DEGs (**Figure 1C**). Clinical information for these 22 IBC samples is provided in **Supplementary Table 4**. Hub genes were selected by intersecting the 90 immune infiltration-related genes from the brown module with the 120 DEGs. This integrated analysis identified *GZMB* and *C16orf54* as candidate hub genes (**Figure 1D**).

### Prognostic value of *GZMB* expression in IBC

**Figure 2:**
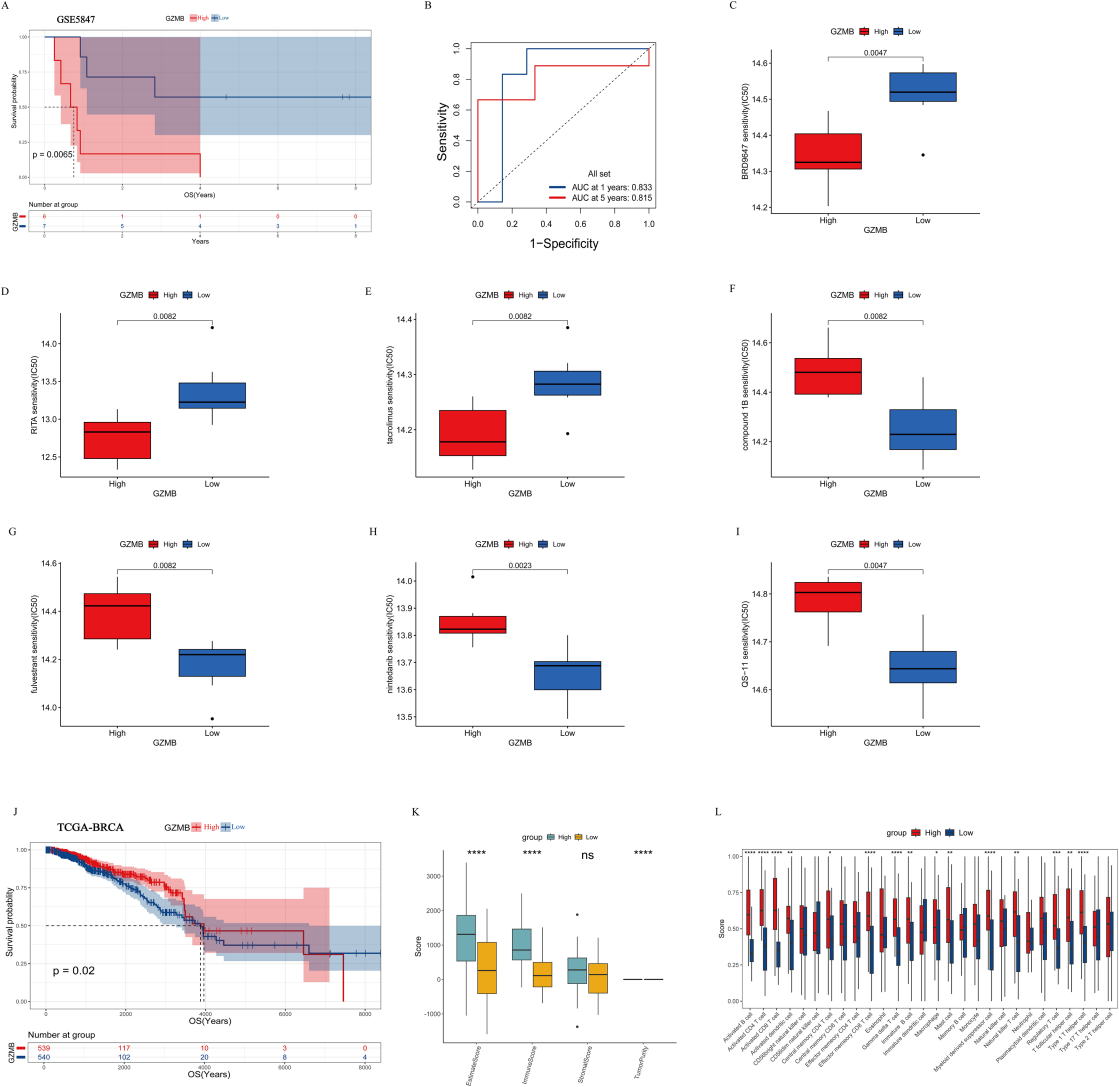
Prognostic value of *GZMB* expression in IBC. **(A)** Kaplan–Meier curves comparing OS between patients with high versus low GZMB expression from GSE5847 dataset. **(B)** Time-dependent ROC curves evaluating the predictive performance of *GZMB* expression for 1- and 5-years survival. **(C–I)** Box plots comparing the estimated IC50 of BRD9647, RITA, tacrolimus, compound 1B, fulvestrant, nintedanib, and QS-11 between *GZMB*-high and *GZMB*-low expression groups. **(J)** Kaplan–Meier curves comparing OS between patients with high versus low *GZMB* expression from TCGA-BRCA dataset for independent cohort validation. **(K)** Comparison of immune scores, estimated using the ESTIMATE algorithm, between *GZMB* expression groups. *p < 0.05, **p < 0.01, ***p < 0.001, ****p < 0.0001. **(L)** Box plot of immune infiltration levels via ssGSEA stratified by *GZMB* expression.

To assess the prognostic value of the identified hub genes, study analyzed a cohort of 13 pre-treatment IBC samples from the GSE5847 dataset, for which survival information was available. Due to the absence of RNA-seq data for *C16orf54* in this cohort, subsequent analyses focused solely on *GZMB*. Patients were stratified into *GZMB*-high (n=6) and *GZMB*-low (n=7) expression groups based on the median expression value. Kaplan-Meier analysis revealed that high *GZMB* expression was significantly associated with poorer OS (P<0.05) (**Figure 2A**). Time-dependent ROC analysis demonstrated strong predictive performance of *GZMB* expression, with area under the curve values of 0.833 and 0.815 at 1 and 5 years, respectively (**Figure 2B**). Furthermore, study explored the association between *GZMB* expression and drug sensitivity using inhibitor response profiling. Patients with high *GZMB* expression showed increased sensitivity to several compounds, including BRD9647, RITA (an inhibitor of p53-MDM2 interaction), and tacrolimus (a calcineurin inhibitor) (**Figures 2C–I**). It should be noted that these drug sensitivity profiles are computational predictions derived from in silico modeling, providing hypotheses for future experimental testing rather than validated therapeutic recommendations. To assess the generalizability and specificity of this finding, we analyzed *GZMB* expression in a large, non-IBC-specific breast cancer cohort (TCGA-BRCA, n=1079). In contrast to IBC, high *GZMB* expression in this general cohort was significantly associated with favorable prognosis (p < 0.05, **Figure 2J**). This inverse association underscores the distinct role of *GZMB* within the unique immune context of IBC. To validate the relationship between *GZMB* expression and immune infiltration, study integrated and batch-corrected 79 pre-treatment IBC samples from four independent datasets (GSE5847, GSE17907, GSE22597, and GSE45581) (**Supplementary Figure 1**). The combined cohort was dichotomized into *GZMB*-high (n=39) and *GZMB*-low (n=40) groups, and the *GZMB*-high group exhibited significantly elevated immune scores (P<0.05) (**Figure 2K**). However, the *GZMB*-high group also displayed a higher abundance of immunosuppressive cell populations, including regulatory T cells, myeloid-derived suppressor cells, and macrophages (**Figure 2L**). These results suggested that *GZMB* overexpression in IBC might contribute to an immunosuppressive tumor microenvironment conducive to immune escape, thus resulting poor prognosis.

### scRNA-seq data processing and cell type annotation

**Figure 3:**
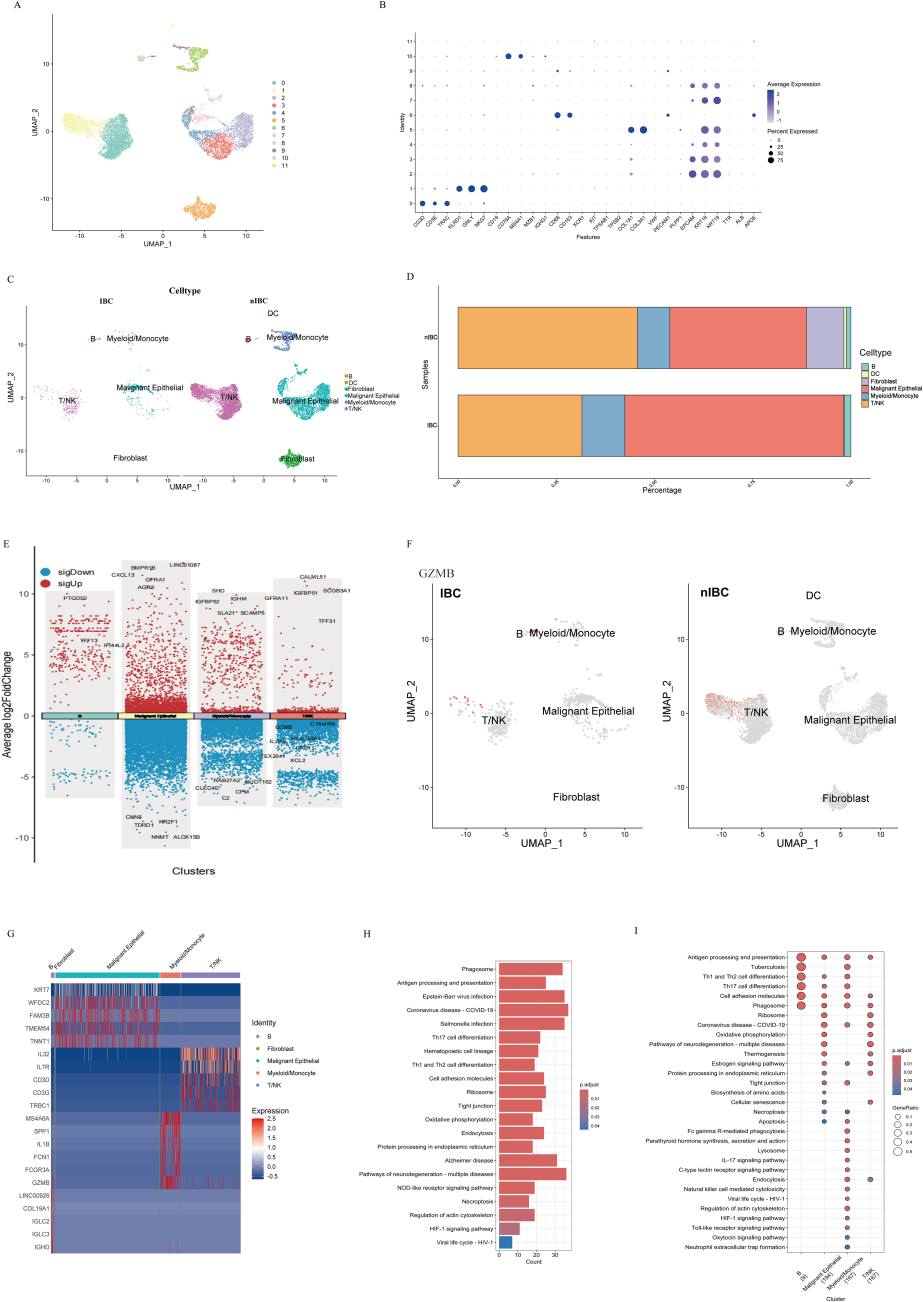
Single-cell atlas of IBC and non-IBC samples. **(A)** UMAP plot of 12 distinct cell subclusters. **(B)** Dot plots depicting average expression of known markers in indicated cell clusters. **(C)** UMAP plots of cells from IBC and non-IBC samples showing 12 clusters in each plot. **(D)** Bar plots depicting the cellular composition of IBC and non-IBC samples across six major cell types. **(E)** Volcano plot of DEGs between IBC and non-IBC samples for each cell type. **(F)** UMAP plot of GZMB expression across integrated single cells from IBC and non-IBC samples. **(G)** Heatmap showing the cell-type-specific top DEGs in IBC. **(H)** KEGG analysis of the single cell global differentially expressed genes in IBC. **(I)** Functional annotation of cell populations in IBC.

To characterize the cellular composition and functional states of the immune microenvironment in IBC, study analyzed scRNA-seq data from 1 IBC sample and 2 non-IBC samples (GSE208532). After initial quality control, retaining genes detected in at least three cells and cells expressing between 200 and 5000 genes, study further excluded cells with mitochondrial gene content exceeding 10% (**Figure 3A**). Although limited by the availability of IBC samples, our analysis profiled 18834 high-quality single cells, enabling a detailed initial characterization of the IBC TME. Cell types were annotated based on established marker genes (**Figure 3B**), yielding 6 major cell populations: B lymphocytes, DCs, fibroblasts, malignant epithelial cells, myeloid/monocytes, and T/NK cells (**Figure 3C**). Comparative analysis revealed a significantly higher proportion of malignant epithelial cells and a corresponding decrease in immune cells in IBC compared to non-IBC samples, indicating a more aggressive tumor phenotype and an immunosuppressive microenvironment in IBC (**Figure 3D**). Myeloid/monocytes constituted the predominant immune population in IBC and were present at a higher frequency than in non-IBC samples. Expression levels of *GZM*B and *C16orf54* were downregulated in T/NK cells from IBC relative to non-IBC (**Figure 3E**). The downregulation of *GZMB* and *C16orf54* in T/NK cells may indicate impaired cytotoxic function, consistent with an immune-evasive microenvironment in IBC. To further investigate the cellular origin of key signature in IBC identified in our bulk analysis, we examined the expression of GZMB across the single-cell atlas. UMAP visualization revealed that *GZMB* expression was predominantly localized to subsets within the myeloid/monocytes populations in IBC, with notably higher expression levels in these clusters compared to B lymphocytes, fibroblasts or malignant epithelial cells (**Figure 3F**). This pattern suggests that myeloid-lineage cells are a major source of *GZMB* within the IBC tumor microenvironment. DEGs across each cell subpopulation in IBC are summarized in **Figure 3G**. Notably, expression levels of *GZMB* and *SPP1* were upregulated in myeloid/monocytes. To investigate functional heterogeneity across cell types, study performed KEGG pathway enrichment analysis. Significantly enriched pathways were associated with intercellular communication, metabolic processes, and cellular transport, including cell adhesion, tight junction, phagosome, and endocytosis pathways (**Figure 3H**). Myeloid/monocytes and malignant epithelial cells exhibited pronounced enrichment in tight junction and apoptosis pathways (**Figure 3I**), suggesting intense crosstalk between these populations that may promote apoptosis and facilitate tumor invasion.

### *SPP1* signaling is associated with an immunosuppressive microenvironment in IBC

**Figure 4:**
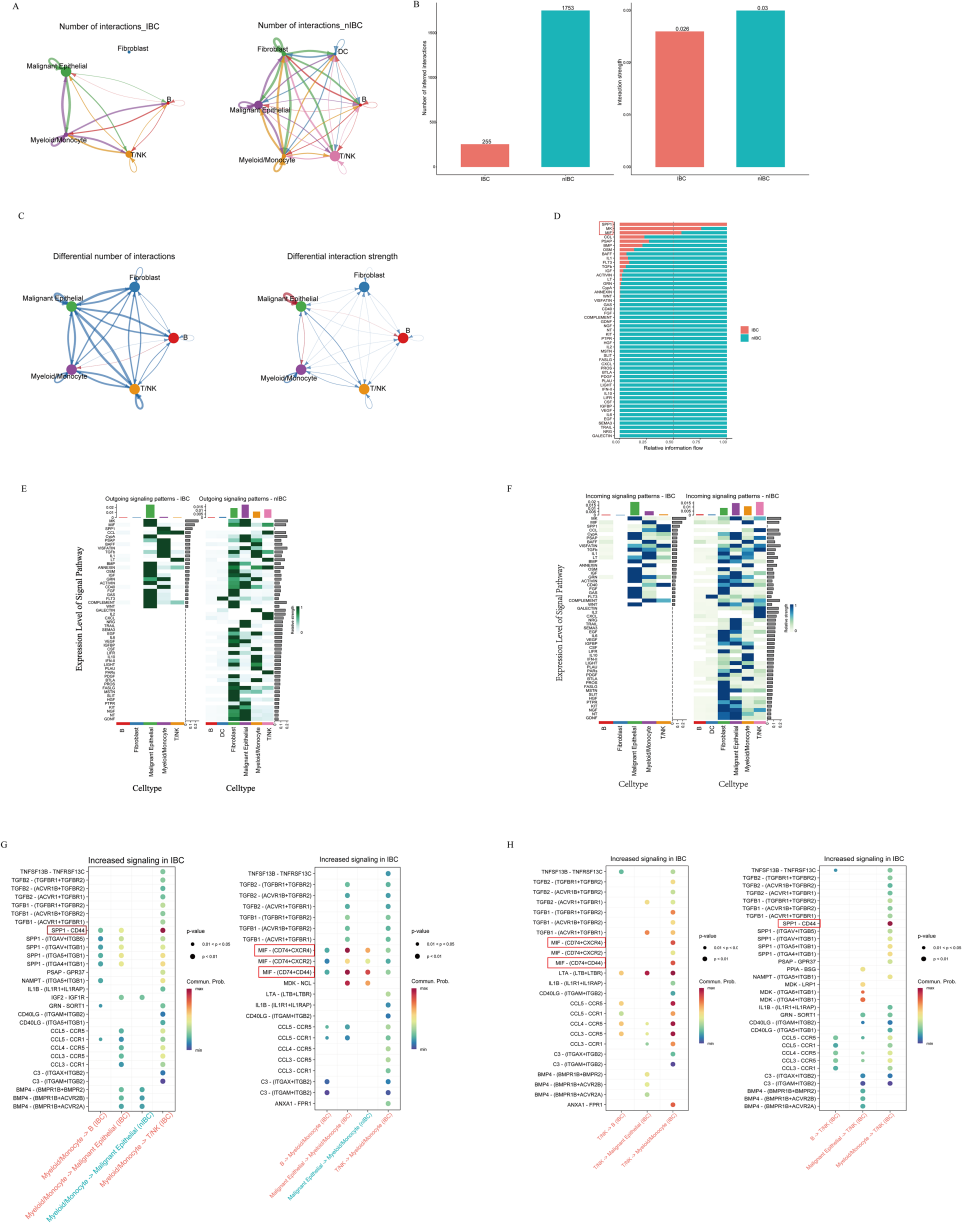
Comparison of cellular communication between IBC and non-IBC samples. **(A)** Cellular communication networks in IBC and non-IBC. Nodes represent cell types. Edges (lines) between nodes represent inferred interactions, with thickness scaled to the total communication probability (thicker indicates more communication). Arrows depict signaling directionality. **(B)** Bar graph showing the total number (left) and interaction strength (right) of ligand – receptor pairs across IBC and non-IBC samples. **(C)** Differentially regulated number and strength of cellular communication network in IBC compared to non-IBC. The color of the lines indicates the direction of change: red signifies communication upregulated in IBC, while blue signifies communication downregulated in IBC. The thickness of the lines indicates the degree of regulation. **(D)** Comparative profiles of pathway signal intensities indicating specific signaling pathways in IBC and non-IBC samples. **(E, F)** Heatmaps displaying expression level of efferent **(E)** and afferent **(F)** signaling pathways across cell types. **(G, H)** Upregulated signaling pathways with myeloid/monocytes as sender (left) and receiver (right) populations in IBC **(G)**. Upregulated signaling pathways with T/NK cells as sender (left) and receiver (right) populations in IBC **(H)**. Red boxes highlight the significantly upregulated signaling pathways in IBC.

To interrogate cell-cell communication within the TME, study utilized the CellChat package to analyze the scRNA-seq data of IBC and non-IBC samples. A comprehensive communication network was constructed to delineate alterations in signaling pathways between IBC and non-IBC samples (**Figure 4A**). Visual comparison reveals that the communication network in non-IBC samples is markedly more active and complex than in IBC, with notably stronger baseline signaling observed across multiple cell types. This contrast highlights a potential suppression or rewiring of cell-cell communication in the IBC microenvironment, prompting us to perform a detailed, pathway-centric analysis to identify specific altered signals. Overall, both the number and strength of cellular interactions were reduced in IBC compared to non-IBC samples (**Figure 4B**). However, in IBC, the number of interactions from B cells to myeloid/monocytes was increased, and the communication strength from malignant epithelial cells to other malignant epithelial cells, myeloid/monocytes, and B cells was significantly enhanced relative to non-IBC samples (**Figure 4C**). Myeloid/monocytes and malignant epithelial cells emerged as central hubs in the cellular communication network in IBC. Comparative analysis of communication probabilities revealed global differences in information flow between IBC and non-IBC. Notably, signaling flows mediated by *SPP1*, *MK*, and *MIF* were markedly amplified in IBC (**Figure 4D**). Heatmap analysis further identified a specific enhancement of *SPP1* signaling from myeloid/monocytes to T/NK cells in IBC, which was nearly absent in non-IBC samples (**Figures 4E, F**). Ligand-receptor analysis indicated that myeloid/monocytes preferentially communicated with T/NK cells via the *SPP1*-*CD44* pair in IBC (**Figures 4G, H**). Additionally, *MIF*- (*CD74* + *CD44*) and *MIF*- (*CD74* + *CXCR4*) signalings were augmented between malignant epithelial cells and myeloid/monocytes, as well as between T/NK cells and myeloid/monocytes in IBC samples (**Figures 4G, H**). Collectively, these results suggest that the malignant progression of IBC may be driven in part by activation of the *SPP1*-*CD44* signaling axis.

### Characterization of distinct myeloid/monocytes subpopulations in IBC

**Figure 5:**
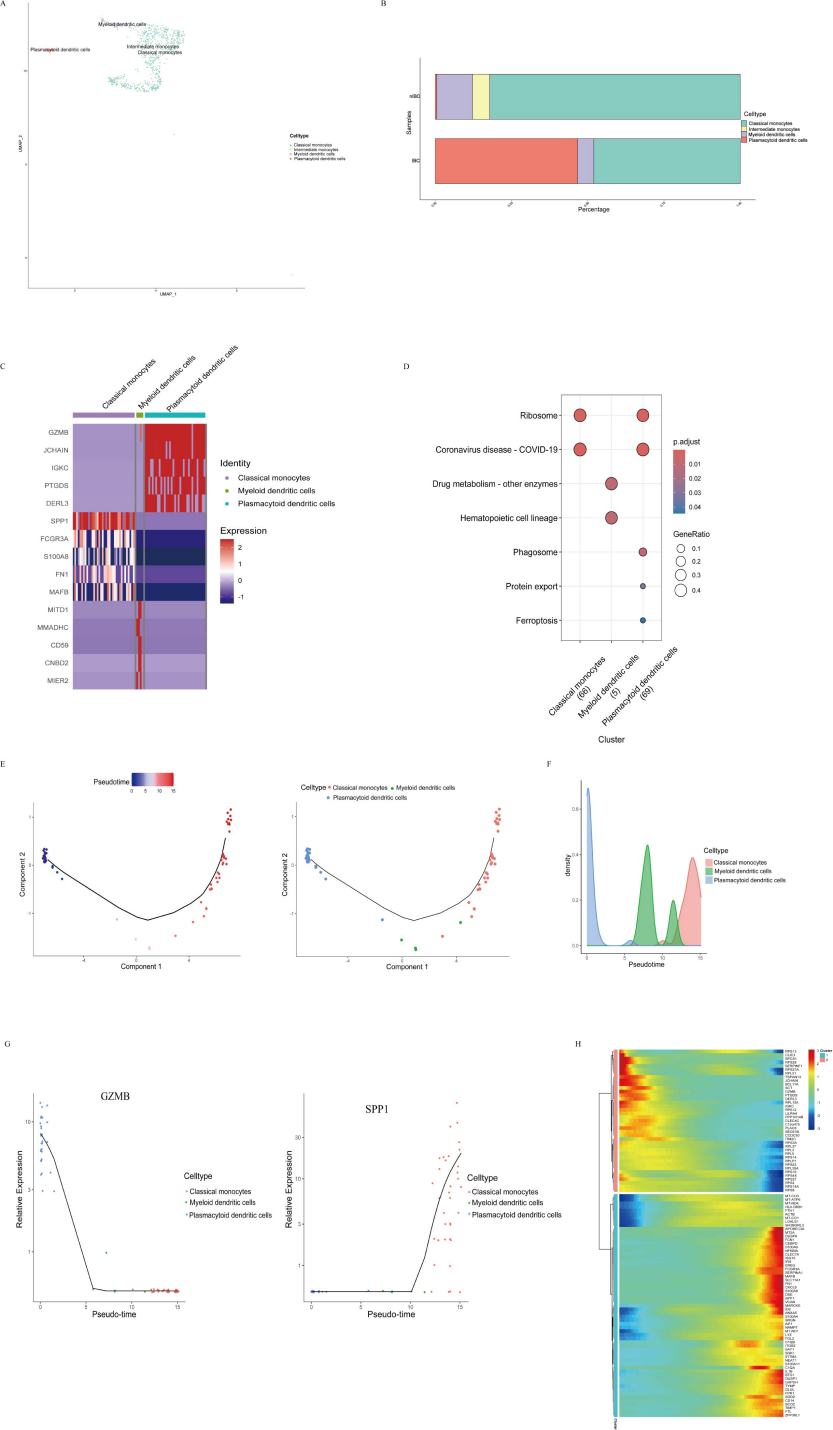
Characterization of myeloid/monocytes subpopulations in IBC. **(A)** UMAP plot of 4 distinct myeloid/monocytes subpopulations. **(B)** Compositional distribution of distinct myeloid/monocytes subpopulations across IBC and non-IBC samples. **(C)** Heatmap of DEGs across cell subpopulations in IBC. **(D)** Dot plot displaying KEGG pathways enriched in each subpopulation. **(E)** Developmental trajectory of myeloid/monocytes subpopulations inferred by pseudotime analysis, colored by pseudotime (left) and cell type (right). The blue point in left plot indicates the start of pseudotime, with warmer colors denoting more advanced differentiation states. **(F)** Density distribution of cells along the pseudotime trajectory. **(G)** Expression dynamics of *GZMB* (left) and *SPP1* (right) across pseudotime. **(H)** Heatmap of gene expression patterns clustered by pseudotime.

Building on prior analyses indicating a central role for myeloid/monocytes in cellular communication within IBC, study further dissected their heterogeneity by performing subclustering analysis. UMAP identified 4 distinct myeloid/monocytes subpopulations (**Figure 5A**). Notably, pDCs were almost exclusively detected in IBC samples (**Figure 5B**). It should be noted that pDCs, a rare yet crucial immunomodulatory cell type, were represented by a relatively small number of cells (approximately 39) in this dataset. Therefore, this study did not perform independent differential expression analysis or make quantitative inferences for low-abundance populations such as pDCs. Their inclusion in the cellular atlas aims to provide a comprehensive census of the immune composition within this cohort and to establish a basis for their potential involvement in subsequent cell-cell communication network analyses. A heatmap of DEGs across these subpopulations is presented in **Figure 5C**, revealing upregulated *GZMB* expression in pDCs and upregulated *SPP1* expression in classical monocytes. This pattern identifies pDCs as a predominant cellular source of *GZMB* within the IBC tumor microenvironment. KEGG pathway enrichment analysis of these subpopulations showed that IBC-specific pDCs were significantly enriched in ribosomal pathways (**Figure 5D**), suggesting heightened protein synthetic activity. To reconstruct the developmental hierarchy within the myeloid/monocytes subpopulations in IBC, study performed pseudotime trajectory analysis. The resulting model suggests a differentiation path wherein immune progenitors initially give rise to pDCs, which subsequently transition into myeloid dendritic cells and ultimately differentiate into classical monocytes (**Figures 5E, F**). Gene expression dynamics along this trajectory showed high *GZMB* levels in early-stage pDCs and elevated *SPP1* in terminal classical monocytes (**Figures 5G, H**).

### Exploratory assessment of *GZMB* and *SPP1* protein expression by immunohistochemistry

As a preliminary exploration, we performed IHC for *GZMB* and *SPP1* on pre-treatment core-needle biopsy samples from a limited cohort of 3 IBC patients who received NAT at our institution. The analysis showed that *GZMB* protein was predominantly localized to the nuclei of immune cells, while *SPP1* was primarily expressed in the cytoplasm of immune cells, malignant epithelial cells, and fibroblasts (**Supplementary Figure S2A**). Quantitative evaluation suggested that baseline protein levels of both *GZMB* and *SPP1* tended to be higher in the two patients with RD compared to the single patient achieving pCR (**Supplementary Figures S2B, C**). No statistical comparison was performed within the RD group due to the minimal sample size. These observations, while derived from a very small sample set, provide initial histopathological support for the potential association of *GZMB* and *SPP1* with therapy resistance in IBC, warranting validation in larger cohorts.

## Discussion

IBC is characterized by highly aggressive progression and a propensity for metastasis. Accumulating evidence indicates that the TME is a pivotal determinant of the aggressiveness and poor prognosis associated with IBC, with significant perturbations in immune homeostasis frequently observed in IBC samples (18–20). Nonetheless, the specific cellular components of the TME and the molecular mechanisms driving these immune alterations remain inadequately characterized. To address this, study employed integrated bulk and single-cell transcriptomic profiling to identify TME-related biomarkers and distinct cell subpopulations, and to investigate the mechanisms underlying TME remodeling in IBC.

Our study identified *GZMB*, an immune infiltration-related gene, as a predictor of survival in IBC. Patients exhibiting high *GZMB* expression experienced poorer clinical outcomes, and *GZMB* expression demonstrated favorable predictive accuracy for 1- and 5-years survival. Although *GZMB* is a serine protease produced by cytotoxic lymphocytes and natural killer cells, typically associated with target cell apoptosis and considered a marker of anti-tumor immunity (21–24), its elevated expression in IBC correlated with immunosuppressive cell activation and an adverse prognosis. Notably, high expression of *GZMB* in pDCs of IBC and its association with immunosuppressive markers suggest a potential non-canonical role in immune regulation. The stark contrast between its correlation with poor prognosis in IBC and favorable prognosis in general breast cancer (TCGA data) underscores the context-dependent nature of its function. This paradoxical observation suggests a model wherein *GZMB* may associated with IBC aggressiveness and immunosuppression, with potential mechanisms involving the induction of immune cell apoptosis and activation of immunosuppressive populations, thereby associating it with TME remodeling. Detailed mechanistic studies are required to elucidate these functions.

Single-cell analysis found that IBC samples exhibited a significantly higher proportion of malignant epithelial cells and a reduced immune cell presence, indicative of an immunosuppressive TME. Although IBC presents with clinical signs of inflammation, the proportion of immune cells within the tumor microenvironment may be lower than that of malignant epithelial cells in some subtypes or samples, suggesting that immunosuppression may be linked to local secretion of inflammatory mediators rather than to absolute immune cell numbers. Myeloid/monocytes constituted the predominant immune compartment in IBC, with elevated expression of both *GZMB* and *SPP1*. *SPP1*+monocytes display M2-like macrophage characteristics associated with reduced antitumor activity, promotion of tumor angiogenesis and enhanced tumor progression (25), which have been linked to immunosuppression and poor prognosis in other malignancies, such as head and neck squamous cell carcinoma (26). In addition, previous study has established that *SPP1* promotes tumor cells proliferation through *PI3K*/*Akt* signaling pathway in breast cancer (27). Cellular communication analysis revealed intense interactions between myeloid/monocytes and malignant epithelial cells in IBC, suggesting their central role in modulating the TME. Study further identified an IBC-specific signaling axis involving *SPP1*-*CD44* directed from myeloid/monocytes to T/NK cells. *SPP1*-*CD44* axis could promote tumor invasion and foster an immunosuppressive microenvironment through T cell inhibition (28, 29). Our cellular communication analysis revealed that in IBC, the observed interaction between *SPP1*+monocytes and exhausted T cells via the *SPP1*-*CD44* axis is associated with an immunosuppressive TME. *MIF* signaling was as well as augmented in IBC samples. Previous studies have established that *MIF* signaling activates the *PI3K*/*Akt* pathway, inhibiting apoptosis and promoting angiogenesis (30). It is important to note that our inference of SPP1-mediated (and other) intercellular communication is derived from computational prediction (CellChat analysis). While this method robustly identifies statistically over-represented ligand-receptor interactions, it does not confirm directional signaling or biological function. Thus, our findings imply an association between these signaling networks (e.g., *SPP1*-*CD44*) and an immunosuppressive context in IBC, rather than proving causation. Future studies employing functional assays are required to validate their precise roles in shaping the IBC immune landscape. Subclustering of myeloid/monocytes uncovered a significant expansion of pDCs in IBC and upregulated *GZMB* expression in pDCs. Prior study has found that pDCs promote the expansion of *Foxp3+*regulatory T cells via the *ICOSL*-*ICOS* axis in ovarian cancer, thereby mediating immunosuppression and facilitating tumor progression (31). Notably, a previous study has suggested that human pDCs can directly deliver *GZMB* protein to T cells and suppress T cell proliferation in a cell-contact-dependent and perforin-independent manner (32). Based on this observation, the high expression of *GZMB* in IBC pDCs may reflect its non-canonical functions or suggest its role in cooperating with other immunoregulatory molecules to foster an immunosuppressive microenvironment. The specific mechanisms require further functional validation. In summary, our study suggests that pDCs with high *GZMB* expression may serve as an initiating factor in the immunosuppressive reprogramming of the TME in IBC. Immunohistochemical staining results supported the potential association of *GZMB* and *SPP1* with therapy resistance in IBC.

This study has several limitations. First, due to the rarity of IBC and limited publicly available datasets, we integrated several cohorts with small sample sizes without uniform baseline characterization. Consequently, potential biases may have been introduced due to differences in patient populations, sample processing procedures, and incomplete clinical annotations. Consequently, the primary findings of this study require validation in future prospective cohorts with comprehensive clinical annotation. Furthermore, the single-cell atlas, while providing a valuable discovery resource, is derived from a limited number of samples. It should be noted that within this atlas, certain rare but important immune cell types, such as pDCs, were represented by a low number of cells. Therefore, findings pertaining to these low-abundance populations are descriptive and hypothesis-generating. Although key discoveries—including the identification of pDCs and the *SPP1*-*CD44* axis as components of the IBC immunosuppressive niche—are supported by our bulk transcriptomic and IHC analyses, prospective validation using multicenter resources and larger scRNA-seq cohorts is warranted to confirm and generalize these observations. The immunohistochemical validation of *GZMB* and *SPP1* protein expression was performed on a very small sample (n=3) and should be considered preliminary. Although the results are consistent with our transcriptomic findings, future studies with larger, well-annotated IBC tissue cohorts are essential for definitive clinical validation. Second, the most significant limitation is the correlative nature of our findings, which lack direct functional experimental validation to establish causality. The absence of robust IBC-specific cell and animal models constrained our ability to test the mechanistic roles of *GZMB* and *SPP1*, or the function of pDCs. Future efforts should focus on developing such models based on the molecular phenotypes identified here to facilitate definitive mechanistic studies. To address this gap, future research plans include: *In vitro* experiments: Overexpression or knockdown of *GZMB* or *SPP1* in IBC cell lines or primary cells to observe their effects on tumor cell proliferation, invasion, apoptosis, and the function of co-cultured immune cells (such as T cells and pDCs). *In vivo* experiments: Utilizing humanized or immunocompetent IBC mouse models to evaluate the effects of *SPP1* knockout or anti-*SPP1* neutralizing antibodies, as well as depletion of pDCs or inhibition of their *GZMB* activity, on tumor growth, metastasis, and treatment response. Mechanistic investigation: Using the above models to further elucidate the downstream signaling pathways of the *SPP1*-*CD44* axis (e.g., *PI3K*/*Akt*) and how *GZMB* protein derived from pDCs specifically affects T-cell function. Only after completing such functional validations can the efficacy of *GZMB* and *SPP1* as therapeutic targets in IBC be firmly established, and conclusive evidence be provided for our proposed model of immunosuppressive microenvironment remodeling. Third, we did not extensively address the impact of IBC molecular subtypes on TME composition and remodeling mechanisms, largely due to the lack of complete subtyping information in the integrated public datasets. Given that different subtypes may exhibit distinct biological behaviors, incorporating subtype-specific analyses in future, well-annotated cohorts will be crucial for enhancing the clinical translatability of our findings. Furthermore, potential confounding factors such as treatment history and tumor heterogeneity warrant consideration. Although the bulk RNA-seq samples were obtained prior to systemic therapy, heterogeneity in prior diagnostic biopsies could influence the transcriptional profile. Additionally, both bulk and single-cell sequencing approaches may not fully capture the spatial and genomic heterogeneity inherent in IBC tumors, which could affect the interpretation of microenvironment composition and cell-cell communication networks.

## Conclusion

This study identifies *GZMB* and *SPP1* as key immunosuppressive microenvironment-related prognostic biomarkers in IBC, establishes pDCs as central mediators of immunosuppressive niche remodeling, and offers novel therapeutic perspectives for immunotherapy in IBC.

## Data Availability

The original contributions presented in the study are included in the article/**Supplementary Material**. Further inquiries can be directed to the corresponding author/s.
