# Supplementary material for: Integrative analysis of bulk and single-cell transcriptomics identifies factors related to immunosuppressive microenvironment to predict unfavorable prognosis in inflammatory breast cancer
Source: Front Immunol. 2026 Jan 6;16:1727590. doi: 10.3389/fimmu.2025.1727590 (PMC12816328; doi:10.3389/fimmu.2025.1727590)
Supplement: Supplementary file 1 [file Table1.docx]

**Supplementary Tables**

Supplementary Table 1. The information of including datasets in the study.

| **Datasets** | **Data type** | **Sample information** | **Source website** |
| --- | --- | --- | --- |
| GSE17907 | Expression microarray | 21 IBC samples | https://www.ncbi.nlm.nih.gov/geo/query/acc.cgi |
| GSE207248 | RNA-seq | 22 IBC samples | https://www.ncbi.nlm.nih.gov/geo/query/acc.cgi |
| GSE5847 | Expression microarray | 13 IBC samples | https://www.ncbi.nlm.nih.gov/geo/query/acc.cgi |
| GSE22597 | Expression microarray | 25 IBC samples | https://www.ncbi.nlm.nih.gov/geo/query/acc.cgi |
| GSE45581 | Expression microarray | 20 IBC samples | https://www.ncbi.nlm.nih.gov/geo/query/acc.cgi |
| GSE208532 | scRNA-seq | 1 IBC sample and 2 non-IBC samples | https://www.ncbi.nlm.nih.gov/geo/query/acc.cgi |

Supplementary Table 2. Clinical characteristics of 3 samples from our center used for immunohistochemical staining.

| **Patient ID** | **Age** | **Molecular subtype** | **Clinical stage** | **Grade** | **NAT^1^ regimen** | **NAT cycle** | **Miller-Payne score** | **Sataloff score** |
| --- | --- | --- | --- | --- | --- | --- | --- | --- |
| 1 | 24 | Luminal B（HER2 negative） | cT4dN3bM0 | 3 | TEC^2^ | 6 | 4 | 1 |
| 2 | 26 | Triple negative | cT4dN1M0 | 2 | TEC | 6 | 2 | 2 |
| 3 | 43 | Luminal B（HER2 negative） | cT4dN1M0 | 3 | TEC | 6 | 5 | 1 |
| ^1^NAT=Neoadjuvant therapy  ^2^TEC=Taxanes+Epirubicin+Cyclophosphamide | | | | | | | | |

Supplementary Table 3. Clinical characteristics of patients from GSE17907 dataset.

| **Characteristic** | **N = 21**^1^ |
| --- | --- |
| **pT** |  |
| pT1 | 2 (9.5%) |
| pT2 | 2 (9.5%) |
| pT3 | 6 (29%) |
| Unknown | 11 (52%) |
| **pN** |  |
| Negative | 5 (24%) |
| Positive | 13 (62%) |
| Unknown | 3 (14%) |
| **Grade** |  |
| 1 | 2 (9.5%) |
| 2 | 2 (9.5%) |
| 3 | 17 (81%) |
| **ER status** |  |
| Negative | 10 (48%) |
| Positive | 11 (52%) |
| **PR status** |  |
| Negative | 11 (52%) |
| Positive | 10 (48%) |
| **HER2 status** |  |
| Positive | 16 (76%) |
| Unknown | 5 (24%) |
| **Ki67 status** |  |
| Positive | 13 (62%) |
| Unknown | 8 (38%) |
| ^1^n (%) | |

Supplementary Table 4. Clinical characteristics of patients from GSE207248 dataset.

| **Characteristic** | **N = 22**^1^ |
| --- | --- |
| **ER status** |  |
| Negative | 12 (55%) |
| Positive | 10 (45%) |
| **HER2 status** |  |
| Positive | 22 (100%) |
| **NAT regimen** |  |
| Dual HER2 blockade+Paclitaxel | 22 (100%) |
| **Response to NAT** |  |
| pCR | 9 (41%) |
| RD | 13 (59%) |
| ^1^n (%) | |

**Supplementary Figures**

**
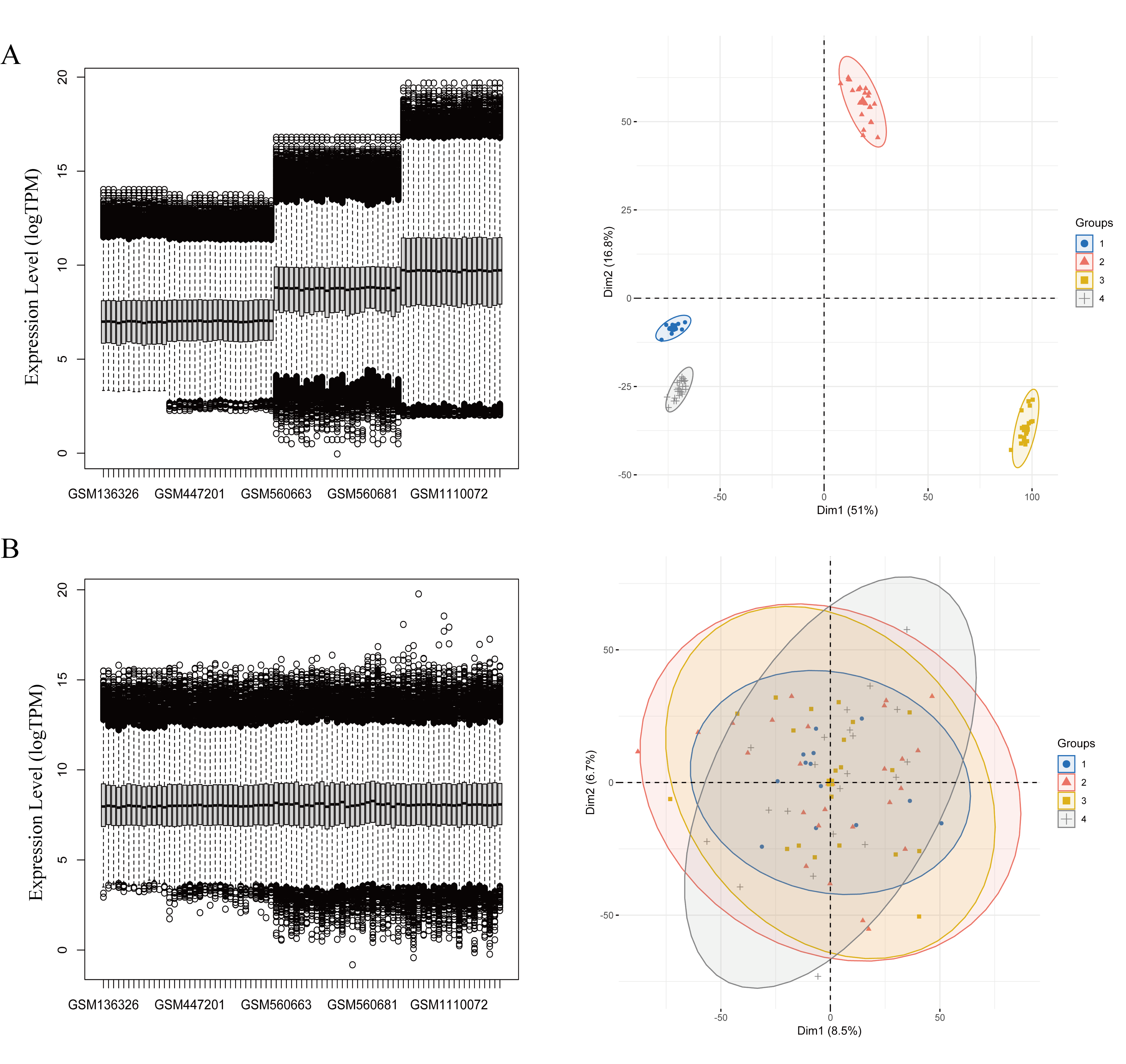
**

**Supplementary Figure 1. (A)** Box plot(left) and PCA plot(right) displaying distribution and clustering of samples before batch effect removal. **(B)** Box plot(left) and PCA plot(right) displaying distribution and clustering of samples after batch effect removal. The box plots (A, left; B, left) show the distribution of data from the merged cohort of 79 samples (GSM accessions). The x-axis labels display a subset of sample IDs due to space limitations.


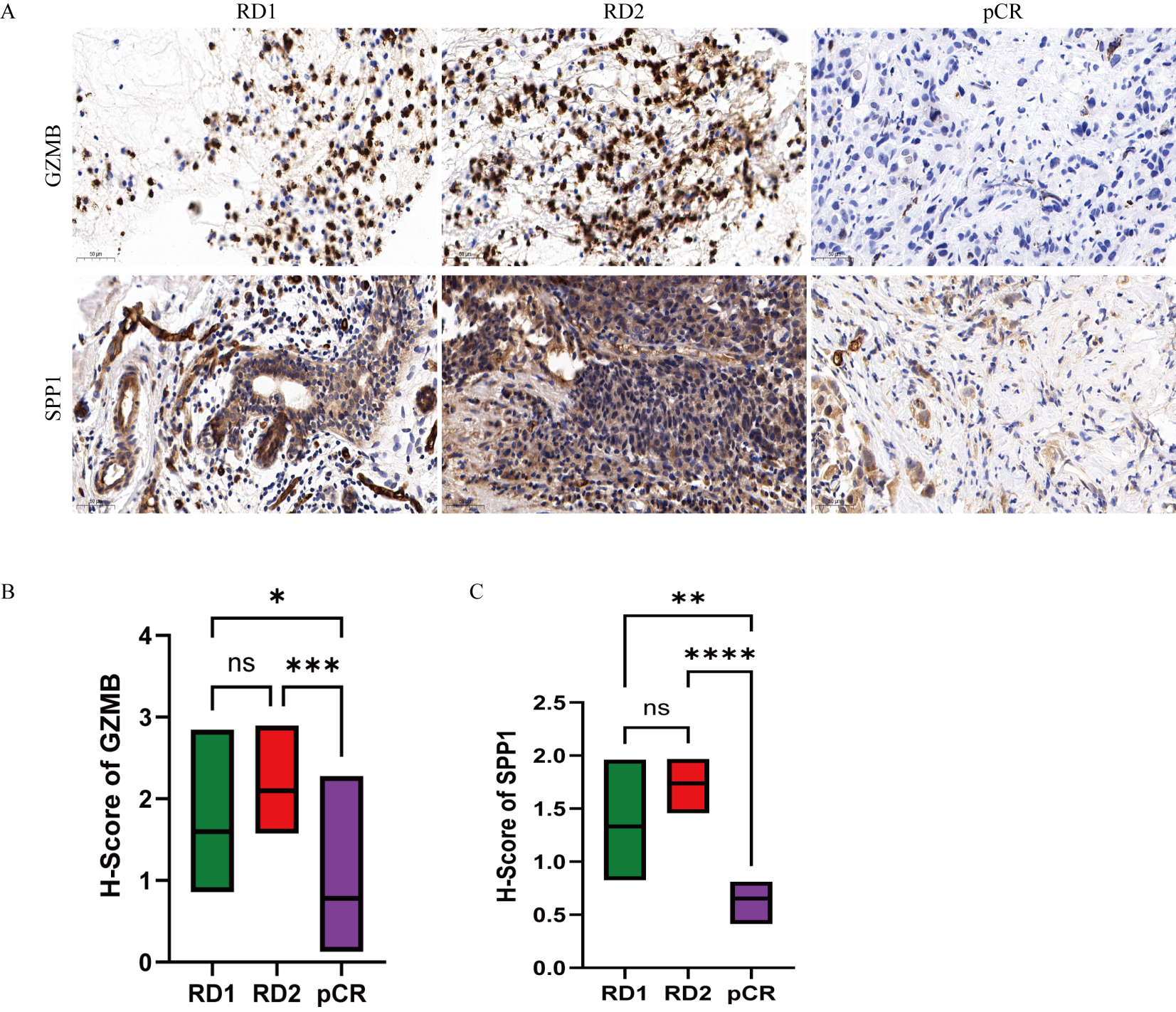


**Supplementary Figure 2.** Exploratory assessment of *GZMB* and *SPP1* protein expression by immunohistochemistry. **(A)** Representative micrographs of *GZMB* and *SPP1* expression within the tumor tissue from 3 patients. **(B)** Box plot showing H-score of *GZMB* protein expression across 3 patients. **(C)** Box plot showing H-score of *SPP1* protein expression across 3 patients.
